# Supplementary material for: Yiai Fuzheng decoction inhibits triple-negative breast cancer by remodeling the immune microenvironment
Source: Front Immunol. 2025 Sep 30;16:1615631. doi: 10.3389/fimmu.2025.1615631 (PMC12518410; doi:10.3389/fimmu.2025.1615631)
Supplement: Supplementary file 2 [file DataSheet1.zip › Supplementary File 2/Treatment/D_vs_H/07_Pathway_Analysis/Pathway_Analysis/Sig_Pathways_ID/Selenoamino acid metabolism.pdf]

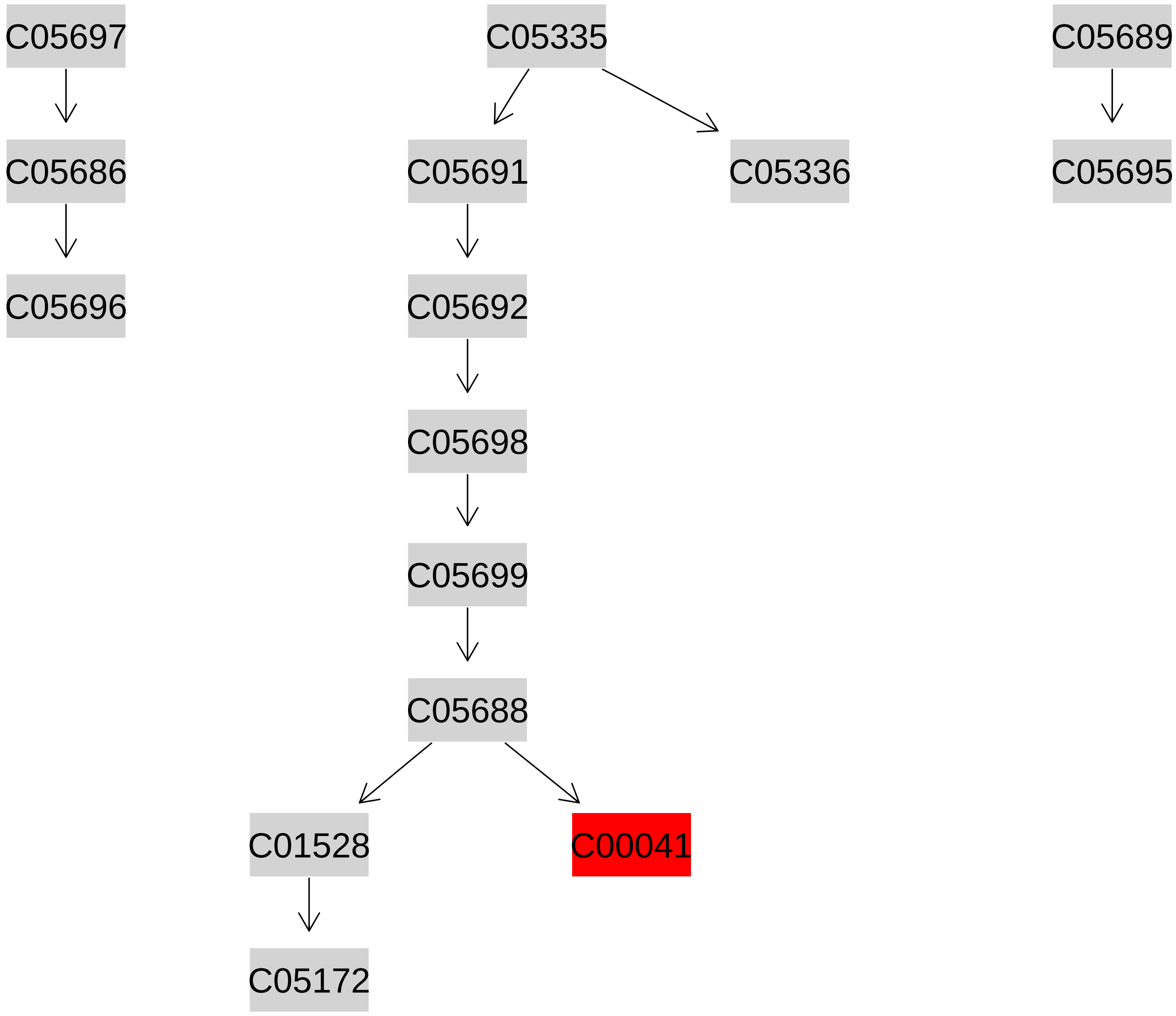
C05697
C05686
C05696
C05335
C05691
C05336
C05692
C05698
C05699
C05688
C01528
C00041
C05172
C05689
C05695
